# Governing brain-computer interfaces as embodied autonomous neural systems: an ethical framework for neurorobotic governance in education

**DOI:** 10.3389/fnbot.2026.1925089

**Published:** 2026-10-16

**Authors:** Jing Gao, Dong Lin, Jianfeng Hu

**Affiliations:** Yantai Institute of Science and Technology, Yantai, Shandong, China

**Keywords:** brain-computer interface, child protection, embodied autonomous systems, governance by design, neuroethics, neurorights, neurorobotics

## Abstract

The rapid development of neurorobotic systems, particularly non-invasive brain-computer interfaces (BCIs) deployed in educational contexts, raises urgent ethical questions about child protection and societal impact. Neurorobotic systems acquire biological signals through embodied sensors, process them via brain-inspired algorithms, and actuate physical responses in closed-loop architectures that directly affect vulnerable learners. We propose Neuro-Adaptive Learning Design (NALD) as a mechanism to translate the capabilities of such neurorobotic systems into ethical safeguards across five functional stages (Sense, Analyze, Decide, Intervene, and Evaluate) that characterize embodied autonomous neural systems. Building on Ethics by Design, we introduce Governance by Design (GbD), incorporating ethical mandates into the technical architecture of neurorobotic systems. The Ethical NALD (E-NALD) framework maps 15 concrete safeguards onto NALD stages, grounded in the Belmont Report and operationalized through the UNESCO 2025 Recommendation on the Ethics of Neurotechnology and the UN Convention on the Rights of the Child. By positioning educational BCIs within the broader context of neurorobotics, this study evaluates the potential societal impact of deploying embodied autonomous systems in compulsory learning environments and offers a governance framework that bridges the gap between abstract neuroethics principles and enforceable technical requirements for neurorobotic technologies. The framework addresses the unique vulnerabilities of children as primary users of neurorobotic systems, including neurodivergent learners who may be simultaneously the most likely beneficiaries and the most at-risk populations.

## 1 Introduction

The use of neuroadaptive technologies in educational settings raises fundamental ethical questions about the protection of children as vulnerable research participants and learners. Consumer-grade electroencephalography (EEG) headsets, previously confined to research laboratories and clinical environments, are now being marketed to schools, parents, and individual learners as tools for attention monitoring, cognitive enhancement, and personalized learning (Ienca et al., 2018). Non-invasive brain-computer interfaces (BCIs), a subset of neurorobotic systems that embody the principles of autonomous neural signal processing and real-time adaptive feedback, have been developed and commercialized by companies such as BrainCo, Muse, NeuroSky, and Neeuro, and have been deployed in classrooms across China, the United States, Europe, and Asia (Ienca et al., 2018; People’s Daily Online, 2019). The commercial market underpinning such deployments is expanding rapidly: the global brain-computer interface market was valued at approximately US$240 million in 2025 and is projected to grow at a compound annual rate of roughly 16%, approaching US$1 billion by 2034 (Fortune Business Insights, 2026). From the perspective of neurorobotics, educational BCIs represent a compelling case of embodied autonomous neural systems: they acquire biological signals (EEG), process them through brain-inspired computational algorithms (machine learning classifiers), and actuate physical responses (adaptive pedagogical interventions) in real-time closed-loop architectures. This positions educational BCIs squarely within the scope of neurorobotic systems as characterized by Knoll et al. (2017)—artificial software and hardware devices that embody neural systems in physical actuation, with direct impact on daily life. We recognize that educational neurotechnologies vary considerably in their degree of autonomy: open-loop attention-monitoring devices perform sensing and analysis without autonomous actuation, whereas closed-loop neuroadaptive systems independently initiate pedagogical interventions and thereby qualify as embodied autonomous neural systems in the full sense; our analysis covers this autonomy spectrum, and the stage-based mapping in Sections 4.2–4.6 applies to whichever stages a given system actually implements. The technical pipeline of neurorobotic educational systems typically involves five subprocesses (Lotte et al., 2018; Zander and Kothe, 2011): (1) signal acquisition through non-invasive EEG electrodes; (2) signal processing and feature extraction using frequency-band analysis; (3) machine learning classification mapping features onto cognitive states; (4) pedagogical decision engines; and (5) continuous evaluation and personalization over time (Zander and Kothe, 2011; Lotte et al., 2018). Educational BCIs constitute neurorobotic systems in the specific sense adopted by this journal (Knoll et al., 2017): they are embodied autonomous systems that integrate neural signal acquisition (Sense), brain-inspired computational processing (Analyze, Decide), and physical actuation (Intervene) within a continuous feedback loop that adapts to the user’s cognitive state in real time. This closed-loop architecture, characteristic of neurorobotic systems, creates governance challenges distinct from those of conventional educational technology. Specifically, the embodied nature of these systems means that they do not merely process information about learners but physically intervene in the pedagogical environment; their autonomy means that interventions may occur without direct human oversight at the moment of actuation; and their closed-loop design means that the system continuously learns from and adapts to neural data, raising concerns about progressive normalization of surveillance and escalating intervention intensity (Williamson, 2019). The Evaluate stage, often under-theorized in conventional technology governance, takes on particular importance in neurorobotic systems because ongoing system adaptation can lead to drift in intervention patterns, accuracy degradation, and emergent behaviors not anticipated at deployment. These neurorobotics-specific considerations motivate our stage-based governance approach, which targets ethical safeguards to the specific technical functions that distinguish neurorobotic systems from other educational technologies. This commercial penetration has significantly outpaced the evolution of sector-specific governance mechanisms, generating a “neurogovernance gap”—the disparity between the sophistication of neurotech capabilities and the capacity of law and ethics to regulate their deployment (Ienca et al., 2018; Ienca and Andorno, 2017). Neuroethicists have articulated abstract principles pertaining to the responsible development of neurotechnology, including cognitive liberty, mental privacy, mental integrity, and psychological continuity (Farahany, 2023), but a research ethics governance system capable of implementing these principles for BCIs used in compulsory learning contexts is not yet in place. A middle-ground mechanism in the form of a “translation process” is necessary to bridge the gap between the conceptual and practical dimensions of neuroethics and technology governance. This governance challenge is especially relevant to neurorobotic systems deployed in educational settings for three related reasons (Organisation for Economic Co-operation and Development, 2019). First, the design of neural interfaces often presupposes a “normative participant” whose sensorimotor and cognitive profile may exclude learners with neurodevelopmental differences, who remain underrepresented in neurotechnology research and development—a recognized equity concern in international responsible-neurotechnology recommendations (Goering et al., 2021). Neurodivergent learners may stand to gain the most from well-designed adaptive technologies while simultaneously facing the greatest risks from poorly designed systems. Second, children and adolescents—the primary participants in educational BCI deployments—are precisely the population for whom international human rights law mandates special protection due to their developmental vulnerabilities and evolving capacities (Lansdown, 2005). The United Nations Committee on the Rights of the Child has emphasized that within digital contexts, children enjoy specific rights not adequately covered by general data protection principles (United Nations Committee on the Rights of the Child, 2021). The UNESCO 2025 Recommendation on the Ethics of Neurotechnology explicitly prohibits the use of such technology for non-therapeutic enhancement of healthy children, but provides limited guidance on national regulatory implementation (United Nations Educational, Scientific and Cultural Organization, 2025). Third, the compulsory nature of schooling creates structural conditions that undermine the assumptions underlying traditional informed consent models (Klein and Ojemann, 2016; Hertz, 2023). A student facing significant academic or social consequences from declining educational obligations cannot meaningfully provide the “free,” “informed,” and “voluntary” consent typically required for legitimate technology use—a problem that debates over freedom of thought and cognitive liberty have brought into sharp focus (Hertz, 2023). This paper addresses this neurogovernance gap by introducing a model for ethical translation called Neuro-Adaptive Learning Design (NALD). The NALD model decomposes an educational BCI system into five functional stages—Sense, Analyze, Decide, Intervene, and Evaluate—each mappable to international law, regional regulation, and comparative governance requirements for neurorobotic systems. We emphasize that the NALD model is offered as a Weberian ideal-type: a deliberate analytical simplification enabling precise ethical targeting by decomposing the “black box” of educational BCIs into analyzable components, rather than an empirical claim about universal system architecture (Weber, 1949). Building on the established “Ethics by Design” (EbD) paradigm, we introduce a novel ethical governance approach termed “Governance by Design” (GbD), which incorporates ethical duties into the technical architecture and institutional governance of neuroadaptive systems. We explicitly position GbD as an extension of existing design approaches—Value-Sensitive Design, Privacy by Design, and regulatory technology (RegTech)—not a conceptual departure from them. The Ethical NALD (E-NALD) framework provides sector-specific guidance for research ethics governance of educational BCIs that accounts for the unique vulnerabilities of children, including those with neurodivergent needs, in compulsory learning contexts. By situating this framework within the broader field of neurorobotics, we demonstrate how governance principles developed for educational BCIs can inform the responsible deployment of embodied autonomous neural systems across diverse application domains.

## 2 From ethics by design to governance by design: theoretical foundations

### 2.1 From value-sensitive design to ethics by design: precursors and boundaries

To situate Governance by Design (GbD), we position it within the lineage of design-based governance approaches. Value-Sensitive Design (VSD) integrates human values into information system design (Friedman and Hendry, 2019; Friedman et al., 2006); Privacy by Design (PbD) operationalizes proactive privacy protection (Cavoukian, 2009); Ethics by Design (EbD) extends this paradigm to broader ethical concerns (Livanis et al., 2024). However, these frameworks differ along two critical dimensions: voluntariness versus enforceability, and scope (design-phase versus lifecycle coverage). The novelty of GbD relative to these precursors lies not in any single element—enforceability exists in the EU AI Act (European Parliament and Council of the European Union, 2024), design-phase ethical coverage in IEEE 7000 (Institute of Electrical and Electronics Engineers, 2021), and technology-assisted compliance in RegTech (Arner et al., 2017)—but in their systematic integration through a functional-stage architecture that maps ethical obligations to identifiable technical functions, enabling stage-targeted governance that none of the precursor frameworks alone provides (Figure 1).

**Figure 1:**
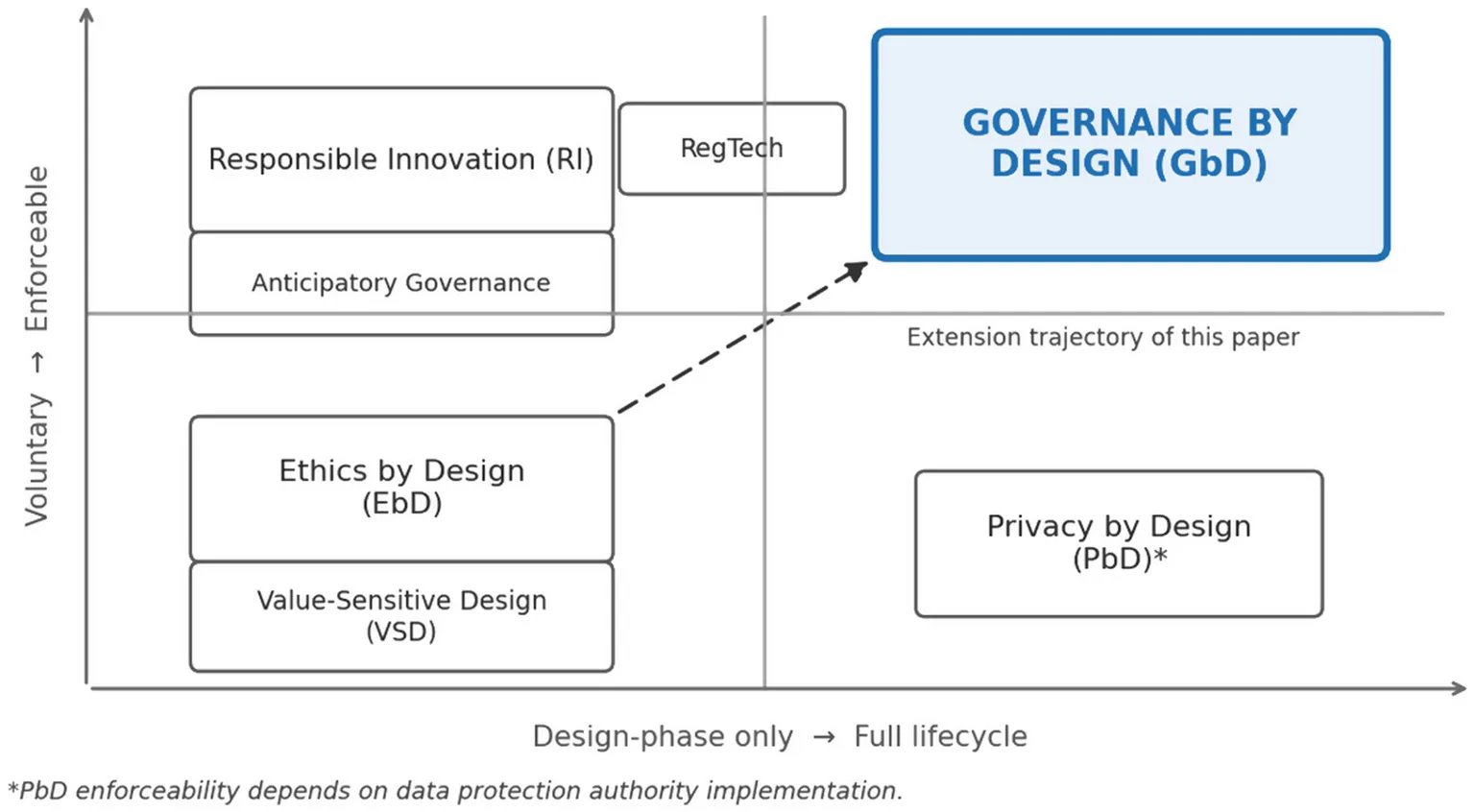
Conceptual positioning of governance by design (GbD). VSD, Value-Sensitive Design; EbD, Ethics by Design; PbD, Privacy by Design; RI, Responsible Innovation; RegTech, Regulatory Technology.

### 2.2 Three limitations of ethics by design

EbD faces three limitations motivating our GbD proposal. First, enforcement mechanisms are largely absent: Hagendorff (2020), Sample et al. (2020), Herrera-Ferrá et al. (2023), and Jobin et al. (2019) have documented that the large majority of AI ethics guidelines lack enforcement mechanisms and that their principles are often too abstract for engineering practice (Schiff et al., 2021). Second, EbD addresses only the design phase, leaving deployment, monitoring, and retirement under-addressed (Hagendorff, 2020). Third, EbD fails to account for sector-specific demands; “fairness” in educational BCIs entails different requirements than “fairness” in loan algorithms (Schermer, 2011). These limitations are particularly consequential for neurorobotic systems, whose embodied nature creates unique governance challenges across the entire technology lifecycle.

### 2.3 Governance by design: a synthesis, not a departure

Governance by Design (GbD) builds on EbD with three additional elements: enforceable ethical standards, lifecycle coverage, and sector-specific operationalization. Its theoretical foundations draw from three literatures. From governance science, we adopt “regulatory technology” (RegTech), examining how technology can facilitate compliance (Arner et al., 2016; Arner et al., 2017), and risk regulation scholarship analyzing how regimes construct and respond to risk (Taeihagh, 2021; Hood et al., 2001). From responsive regulation, we borrow “graduated enforcement”—a spectrum of enforcement options graduated by risk levels (Ayres and Braithwaite, 1992). From anticipatory governance scholarship, we adopt proactive regulatory engagement prior to broad-based deployment (Guston, 2014). We explicitly clarify what GbD does not claim. GbD does not replace or supersede VSD, PbD, or EbD, but rather represents a synthesizing extension building upon these established frameworks while addressing specific limitations in enforcement and operationalization. GbD does not advocate for fully automated governance; human judgment remains essential for contextual safeguard interpretation, novel scenario adaptation, and ensuring ethical compliance serves underlying ethical purposes rather than constituting mere box-ticking. Finally, while GbD-informed policies should not constitute uncritical extensions of the governance state, the proposed framework includes explicit limits and sunset provisions to prevent research ethics governance overreach from stifling beneficial innovation. This synthetic approach holds particular relevance for neurorobotic systems, where uncritical technology adoption has frequently outpaced ethical and governance considerations (Selwyn, 2019; Williamson, 2019; Clausen et al., 2017). Rather than treating ethical governance compliance as a post-deployment afterthought, GbD elevates it to equal status with accuracy and usability as a design consideration for embodied autonomous neural systems.

## 3 The NALD model: an analytical ideal-type for educational BCIs

### 3.1 Neuroadaptive learning: technical architecture and current capabilities

Neuroadaptive learning systems monitor and adapt instantaneously to learner cognitive and behavioral factors to maintain optimal cognitive functioning (Zander and Kothe, 2011). The technical pipeline involves five subprocesses: (1) signal acquisition through non-invasive EEG electrodes embedded in headsets or headbands, sometimes augmented with auxiliary physiological sensors; (2) signal processing and feature extraction using frequency-band power ratios, event-related potentials, or connectivity measures; (3) machine learning classification mapping extracted features onto cognitive state categories such as “focused,” “distracted,” “frustrated,” or “relaxed” (Lotte et al., 2018); (4) pedagogical decision engines mapping estimated cognitive states to instructional actions; and (5) continuous evaluation and personalization over time (Zander and Kothe, 2011). Current technical capabilities exhibit significant limitations. Consumer-grade EEGs typically employ 3–8 channels with dry electrodes providing substantially lower signal quality than research-grade systems (Craik et al., 2019). Classification accuracy under controlled conditions ranges from 60 to 80%, declining further in real-world classroom environments (Lotte et al., 2018; Craik et al., 2019). Thompson (2019) has challenged the framing of “BCI illiteracy”—users’ failure to achieve reliable BCI control—arguing that this framing inappropriately attributes responsibility to users rather than the BCI system itself. Their analysis underscores the governance imperative of developing BCI systems accommodating the full spectrum of neural function, not only populations that perform well in laboratory settings. Systematic performance disparities across demographic subgroups remain a significant challenge for the Bias Audit Rule (Section 4.3), as documented in analogous algorithmic systems (Obermeyer et al., 2019), and pipeline latency (2–10 s) may be too slow for time-sensitive adaptations (Kosmyna and Maes, 2019). While deep learning approaches are promising, performance demands may conflict with on-device processing constraints (Craik et al., 2019). In this technical context, we propose the analytical ideal-type NALD model, comprising five functional stages (Figure 2).

**Figure 2:**
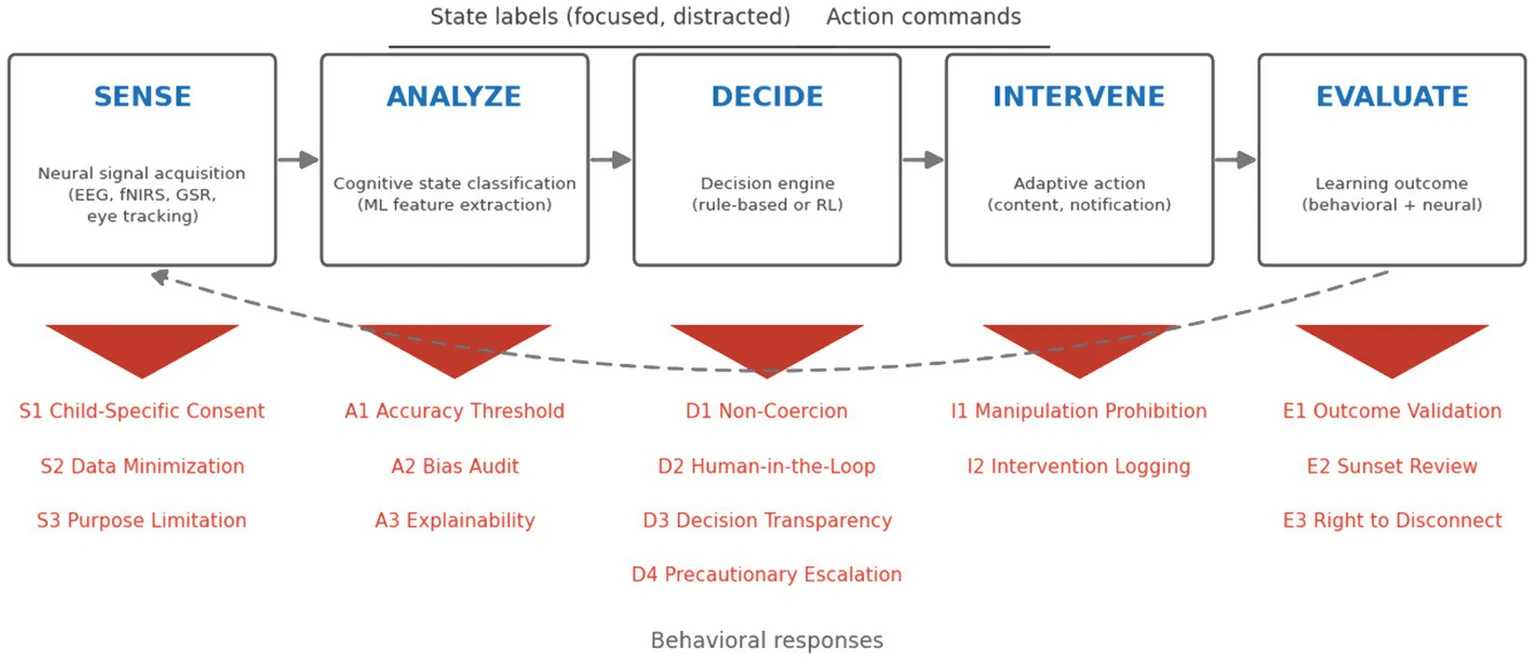
The NALD model: Five-stage technical pipeline (Sense-Analyze-Decide-Intervene-Evaluate) with corresponding E-NALD governance intervention points. fNIRS, functional near-infrared spectroscopy; GSR, galvanic skin response; ML, machine learning.

## 4 Mapping the NALD model onto ethical safeguards: the E-NALD framework

This section presents the paper’s central contribution: a systematic mapping between each NALD stage and concrete ethical safeguards grounded in international instruments and comparative law. For each safeguard, we identify the ethical foundation, implementation requirements, and ethical status (binding ethical standard [B], non-binding ethical guidance [S], or treaty [T] requiring national implementation). The complete E-NALD framework is presented in Table 1. Throughout this section we distinguish three relationships between a safeguard and its source instruments: some safeguards restate existing legal obligations (for example, S2 and S3 apply established GDPR principles to neural data); some operationalize broader rights or principles into technical requirements without themselves constituting direct legal duties; and some—notably A1, A2, D1, and the three-factor test of I1—extend beyond current data protection law as sector-specific propositions justified by children’s heightened vulnerability in compulsory educational settings. The B/S/T status recorded in Table 1 refers to the binding force of the source instrument, not to the safeguard’s independent legal effect.

**Table 1:** The ethical NALD (E-NALD) framework: complete mapping of NALD stages to research ethics governance safeguards.

Stage	Safeguard	Status	Description	Legal basis
Sense	S1 Child-Specific Consent	T	Dual-track parental consent + child assent	UN CRC Art.12
Sense	S2 Data Minimization	B	On-device processing; collect only necessary features	GDPR Art.5(1)(c)
Sense	S3 Purpose Limitation	B	No commercial profiling or sale to third parties	GDPR Art.5(1)(b)
Analyze	A1 Accuracy Threshold	B	Validated accuracy ≥80% (provisional benchmark) before individual-level use	EU AI Act Art. 15
Analyze	A2 Bias Audit	B	Accuracy disaggregated by neurodevelopmental status; provisional 10-point disparity bound	EU AI Act Art. 10; UN CRC Art. 2
Analyze	A3 Explainability	B	Outputs explainable to non-technical stakeholders	EU AI Act Art.13
Decide	D1 Non-Coercion	S	No punishment/shaming for mental states	UNESCO 2025
Decide	D2 Override Capacity	S	Teacher/student can override algorithmic suggestions	IEEE 7000
Decide	D3 Decision Transparency	B	Decision logic documented and auditable	EU AI Act Art. 13—14
Decide	D4 Precautionary Escalation	T	Sustained negative states escalate to human review	UN CRC
Intervene	I1 Manipulation Prohibition	T	Three-factor test for legitimate pedagogical use	UN CRC Art.3
Intervene	I2 Intervention Logging	B	All system interventions logged retrospectively	GDPR Art. 5(2)
Evaluate	E1 Outcome Validation	T/S	Demonstrable pedagogical benefits via controlled trials	UNESCO 2025; UN CRC Art. 3
Evaluate	E2 Sunset Review	B	Time-limited retention with automatic deletion	GDPR Art.5(1)(e)
Evaluate	E3 Right to Disconnect	T/S	Refuse monitoring without educational consequence	UN CRC Art. 3 & 12; UNESCO 2025

Status: B, hard law (binding); S, soft law (non-binding guidance); T, treaty (creates state obligations requiring domestic implementation). Status indicators refer to the source instrument, not to the independent legal force of the safeguard itself. Legal-basis citations indicate the closest source provision; for example, the EU AI Act Art. Ten citation for A2 refers to its data-governance and representative-data requirements—the closest existing hook for bias auditing—rather than to a dedicated bias-audit clause.

### 4.1 Framework development methodology

Because E-NALD is a conceptual synthesis rather than the report of a primary empirical study, we make the framework’s construction explicit. Sources were selected according to four eligibility criteria: (i) binding international and regional instruments addressing neurotechnology, artificial intelligence, data protection, or children’s rights; (ii) soft-law instruments issued by UN-system organizations and the OECD; (iii) peer-reviewed neuroethics, neurorobotics, and BCI literature; and (iv) publicly available vendor documentation and independent technical reviews, used only for product-level claims (the deployment cost estimates in Section 5.3). We then applied a three-step derivation procedure. First, we decomposed the neuroadaptive pipeline into five functional stages through an inductive synthesis of the technical literature (Lotte et al., 2018; Zander and Kothe, 2011). Second, for each stage we identified the ethical risks most frequently documented in the eligible literature for educational and child-facing deployments. Third, we mapped each identified risk to concrete provisions in the eligible instruments, retaining a candidate safeguard only where an identifiable provision or established principle could be cited as its foundation; the B/S/T status in Table 1 records the binding force of that source instrument.

The comparative judgments in Table 2 were derived by examining each instrument’s text against the 15 safeguards and classifying coverage as full, i.e., explicit coverage (the instrument contains a provision addressing the same function at a comparable level of operational specificity); partial, i.e., implicit or general coverage (the instrument addresses the underlying principle without stage-level operational requirements); or no coverage, i.e., absent. Two limitations follow. First, the framework’s thresholds and benchmarks are propositions for empirical validation, not findings. Second, because source selection prioritized instruments with direct relevance to educational deployment, the framework’s legal anchors are concentrated in EU and international instruments; we discuss the implications of this concentration for Global South contexts in Section 5.3 (Figure 3).

**Table 2:** Comparative positioning of E-NALD against existing governance instruments.

Dimension	UNESCO 2025	EU AI Act	E-NALD
Stage-level governance mapping	Partial	Partial	Full
Child-specific safeguards	Partial	No	Full
Accuracy thresholds	No	Partial	Full
Bias audits (neurodevelopmental)	No	Partial	Full
Explainability requirements	General	General	Sector-specific
Override capacity	No	No	Full
Manipulation prohibition	General	No	Full (3-factor test)
Intervention logging	No	No	Full
Sunset review / right to disconnect	Partial	No	Full
Outcome validation	No	No	Full
Neurodiversity provisions	No	No	Full
Global South adaptation	No	No	Universal floor (phased)

Full indicates explicit coverage; Partial indicates implicit or general coverage; No indicates no coverage. The table compares E-NALD against two existing governance instruments—the UNESCO 2025 Recommendation on the Ethics of Neurotechnology (United Nations Educational, Scientific and Cultural Organization, 2025) and the EU AI Act (European Parliament and Council of the European Union, 2024)—with UNICEF policy guidance on AI and children’s rights (United Nations Children’s Fund, 2024; United Nations Children’s Fund, 2021) used as a supplementary soft-law reference point. This comparison demonstrates that while each instrument addresses aspects of educational BCI governance, none provides the systematic stage-level governance mapping that characterizes the E-NALD framework.

**Figure 3:**
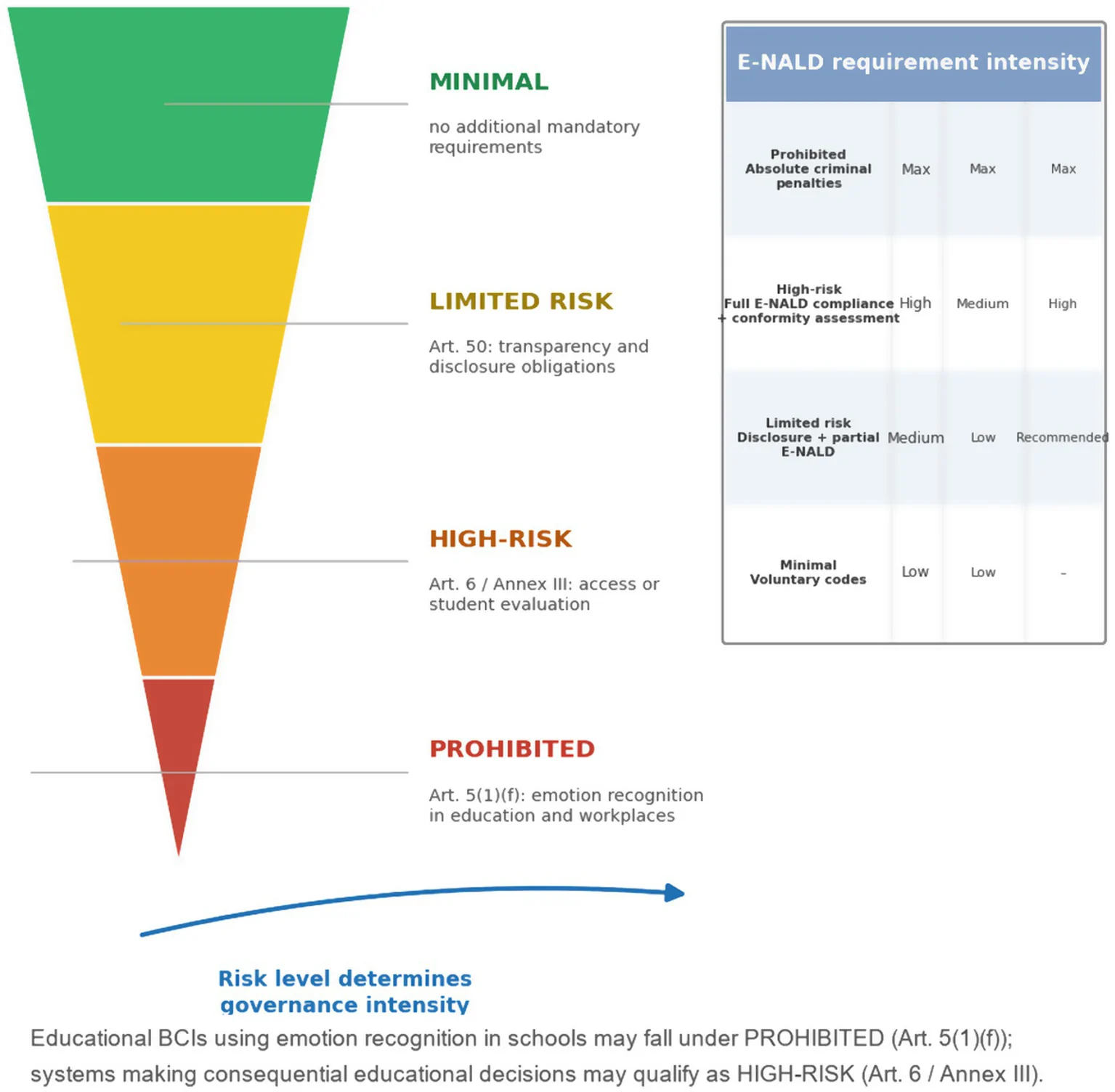
EU AI Act risk classification pyramid for educational BCIs. The vast majority of consumer EEG applications fall into the limited-risk category, but neuroadaptive systems with significant decision-making autonomy may qualify as high-risk under Annex III.

### 4.2 Layer 1: sense stage rules

At the Sense stage, neural signals from learners are captured by electrodes. The principal governance concerns are data sensitivity-neural data constitutes a recognized special category of personal information demanding enhanced protection (Ienca and Malgieri, 2022)-and consent validity (standard informed consent mechanisms are widely acknowledged as problematic for participants under 18). We propose three safeguards for protecting child participants at this stage. Safeguard S1 (Child-Specific Consent): Educational BCI systems should be required to obtain consent through a dual-track mechanism of parental consent and developmentally appropriate child assent, as articulated in Article 12 of the UN Convention on the Rights of the Child (United Nations Committee on the Rights of the Child, 2021). This supplements standard data protection safeguards based solely on parental consent. The dual-track approach acknowledges children as rights holders whose capacities evolve with age (Lansdown, 2005). Assent should explicitly include information for adolescents regarding what neural data is collected, how it is used, and what safeguards are in place. Safeguard A2 (Data Minimization): Only neural features necessary for the stated pedagogical purpose may be collected; raw EEG data should be processed on-device where technically feasible to reduce exposure of sensitive raw signals (Gurses et al., 2015). The GDPR data minimization principle [Article 5(1)(c)] (European Parliament and Council of the European Union, 2016) is operationalized in the neural data context: neural data may inadvertently contain information beyond targeted cognitive states—including emotional responses, fatigue levels, and clinically relevant neurological patterns that neither the learner nor the school would wish to access. This concern relates to the “brain transparency” issue (Ienca et al., 2018)—the possibility that neural signals could reveal information about the learner that even the learner cannot consciously report, such as pre-conscious processing patterns, subclinical emotional states, or implicit biases. A BCI system renders a student’s mental state as “frustrated” or “distracted,” transforming privately experienced states into publicly observable data. Data Minimization reduces risks by maintaining collection scope at the minimum necessary to limit unwanted psychological inference. Safeguard S3 (Purpose Limitation): Neural data gathered for educational purposes cannot be used for commercial profiling, predictive analytics, employment screening, or sale to third parties (United Nations Educational, Scientific and Cultural Organization, 2025; European Commission, 2019; Goering et al., 2017). This safeguard directly addresses ed-tech business models involving sale or sharing of student data with third parties for advertising or other purposes (United Nations Educational, Scientific and Cultural Organization, 2025; Council of Europe, 2024). Neural data presents particularly severe risks in this regard, as it may reveal stable biometric markers that cannot be replaced if compromised.

### 4.3 Layer 2: analyze stage rules

At the Analyze stage, cognitive state is classified from processed neural signals. Central considerations include accuracy requirements for pedagogical decisions, potential bias affecting vulnerable populations, and explainability enabling meaningful human oversight. We propose three safeguards. Safeguard A1 (Accuracy Threshold): Educational BCI classifier accuracy must be established through objective behavioral validation before any deployment decision (Burwell et al., 2017). Consumer EEG devices achieve only 60–80% accuracy in binary cognitive state classification under laboratory conditions, with substantial degradation in real-world environments (Lotte et al., 2018). An accuracy of 65% on a two-class problem represents only a 15-percentage-point improvement above chance (50%)—inadequate for high-stakes pedagogical applications such as identifying students for intervention, modifying assessment modalities, or informing teacher judgments. We therefore propose, as a provisional benchmark rather than a settled governance standard, that educational BCIs demonstrate validated accuracy of at least 80% before being used for individual-level pedagogical decisions. Three caveats define the provisional status of this benchmark. First, accuracy (the overall proportion of correct classifications) must not be conflated with sensitivity, specificity, or psychometric reliability: these metrics capture different performance dimensions, and no single numerical threshold can substitute for context-appropriate validation across all of them. Second, the benchmark is anchored by analogy rather than direct evidence: educational and psychological tests conventionally require reliability greater than 0.80 for valid individual-level decision-making (American Educational Research Association et al., 2014), and decision-support systems in other high-stakes domains are expected to undergo rigorous pre-deployment analytical validation (Morley et al., 2020); educational BCIs make similarly consequential judgments about learners’ cognitive states. Third, performance is population- and setting-specific: a system meeting the benchmark in one population or context may fall below it in another, so governance authorities should set and periodically review context-specific thresholds. Pending such empirical calibration, systems that cannot demonstrate the benchmark should be restricted to developmental or experimental use and must not inform individual-level pedagogical decisions. Safeguard A2 (Bias Audit): Procedures must report and analyze accuracy metrics disaggregated by demographic factors including neurodevelopmental status (Obermeyer et al., 2019). Systems exhibiting accuracy differences greater than 10 percentage points for neurodivergent populations should be remediated or restricted. As with A1, we present this disparity bound as a provisional benchmark whose empirical calibration should be revisited as comparative accuracy data accumulate. This requirement is grounded in Article 10 of the EU AI Act (representative training data) and UN CRC non-discrimination principles (Article 2) (European Parliament and Council of the European Union, 2024; United Nations Committee on the Rights of the Child, 2021). Safeguard A3 (Explainability): Classification outputs must be explainable to non-technical stakeholders including students, parents, and teachers (Khosravi et al., 2022). The EU AI Act Article 13 requires that high-risk AI systems be sufficiently transparent for output recipients to understand their operation. In educational BCI contexts, explainability means stakeholders can understand why a student is labeled “distracted” or “frustrated”—when a BCI output is a probability score from a black-box system, the classification rationale should be expressed in terms of observable signal features and their relationship to the classification (Khosravi et al., 2022; Rudin, 2019).

### 4.4 Layer 3: decide stage rules

The Decide stage involves mapping estimated cognitive states to pedagogical actions via decision engines. Key governance concerns include autonomy preservation—ensuring algorithmic decisions support rather than supplant human pedagogical judgment—and maintaining learner agency over their educational experience. We propose four safeguards. Safeguard D1 (Non-Coercion): Neuroadaptive systems must not function as instruments of punishment or shaming for mental states beyond learner control. Blitz and Bublitz (2021) argue that such uses infringe mental integrity; commentators have raised similar concerns regarding reliance on involuntary mental processes for institutional sanction (Hertz, 2023). The UNESCO 2025 Recommendation expressly prohibits uses that “undermine the autonomy and decision-making capacity of individuals” (United Nations Educational, Scientific and Cultural Organization, 2025). Safeguard D2 (Override Capacity): Teachers and/or students must be able to override, modify, or disregard algorithmically generated pedagogical suggestions. Algorithmic systems should complement human pedagogical judgment, not supplant it. Override capacity must extend beyond a “dismiss” button to enable meaningful influence or reversal of system decisions. Safeguard D3 (Decision Transparency): The logic determining cognitive-to-pedagogical mappings must be documented, inspectable, and auditable, including decision thresholds and machine learning models employed in the decision process. Safeguard D4 (Precautionary Escalation): If the system detects sustained negative cognitive states (e.g., prolonged frustration or distress), it must escalate to human review rather than autonomously intensifying intervention (United Nations Committee on the Rights of the Child, 2021; European Parliament and Council of the European Union, 2024). This prevents counterproductive scenarios where system responses to difficulty indicators inadvertently exacerbate learner distress.

### 4.5 Layer 4: intervene stage rules

At the Intervene stage, pedagogical actions are executed through adaptive content delivery. Primary concerns are preventing manipulation and protecting against emotional or psychological harm. We propose two safeguards. Safeguard I1 (Manipulation Prohibition): Interventions must not exploit identified emotional or cognitive vulnerabilities to influence behavior beyond legitimate pedagogical purposes (Williamson, 2019; Susser et al., 2019). We propose a three-factor test: (a) the intervention targets a cognitive vulnerability identified through neural monitoring; (b) it produces behavior the learner would not choose under reflective conditions; and (c) it serves primarily commercial or institutional interests rather than the learner’s educational goals. All three factors must be present simultaneously (United Nations Committee on the Rights of the Child, 2021; United Nations Children’s Fund, 2021). This test is compatible with diverse normative frameworks: condition (b) reflects the educational goal of developing considered decision-making capacity. Safeguard I2 (Intervention Logging): System-initiated pedagogical interventions must be logged for retrospective review, including triggering cognitive state classification, intervention details, timing, and learner identification (Kosmyna and Maes, 2019; European Parliament and Council of the European Union, 2024; European Parliament and Council of the European Union, 2016). These logs serve multiple governance functions: enabling teachers and parents to monitor system actions on learners; supporting quality assurance and bias audits by recording intervention patterns across learner groups; and providing evidence for complaints or disputes regarding system behavior.

### 4.6 Layer 5: evaluate stage rules

The Evaluate stage assesses intervention effectiveness and enables personalization over time. Key concerns are: ensuring systems are demonstrably linked to learning outcomes; and preventing accumulation of neural profiles that could enable discrimination or manipulation. We propose three safeguards. Safeguard E1 (Outcome Validation): Systems must demonstrate measurable pedagogical benefits through controlled evaluation, not merely technical performance metrics such as classification accuracy (Selwyn, 2019; Williamson, 2019; Clausen et al., 2017). Educational technology has been criticized for deploying systems based on technical capability demonstrations lacking evidence of classroom effectiveness (Selwyn, 2019; Williamson, 2019)—a pattern observed across the neurotechnology space as the “hope and hype” cycle (Clausen et al., 2017). Appropriately designed studies with adequate sample sizes and comparison conditions must demonstrate that neuroadaptive systems produce meaningful improvements in learning outcomes, engagement, or other educationally relevant variables. Safeguard E2 (Sunset Review): Neural data retention must be time-limited with automatic deletion aligned with documented educational value periods; longitudinal profile creation without documented current educational benefit is prohibited (Kosmyna and Maes, 2019; United Nations Children’s Fund, 2024). While learning trajectories may justify data retention over months or years, risks accumulate as educational value diminishes with indefinite retention. Safeguard E3 (Right to Disconnect): Students must have the right to refuse neuroadaptive monitoring without educational consequence (United Nations Committee on the Rights of the Child, 2021; United Nations Educational, Scientific and Cultural Organization, 2025). This safeguard addresses the structural coercion concern: where declining BCI monitoring would result in lost educational opportunities, poor learning experiences, or social stigma, “consent” is not truly voluntary. Schools must provide alternative learning pathways offering equivalent educational quality. The alternative pathway may involve enhanced human support (teacher observation, differentiated instruction) or alternative technology (non-neural sensors such as keyboard dynamics and response-time analysis). Equivalence of educational opportunity, not identity of method, is the governing criterion.

## 5 Implementation pathways and governance challenges

### 5.1 Institutional mechanisms for E-NALD implementation

Implementing E-NALD requires distinguishing four governance contexts that are frequently conflated in current debates: research ethics review of studies involving educational BCIs; public procurement of BCI systems by schools and districts; commercial deployment of BCI products by vendors; and routine school use of already-deployed systems. Each context involves different oversight actors, legal instruments, and points of leverage, and we discuss each in turn.

Research ethics review. For studies that evaluate educational BCIs or generate evidence about their effects, institutional review boards (IRBs) should adopt E-NALD-based review procedures (Fins, 2015; Council for International Organizations of Medical Sciences, 2016; National Commission for the Protection of Human Subjects of Biomedical and Behavioral Research, 1979), including mandatory neurodiversity impact assessments and independent oversight. In this context, the oversight question is whether the research itself meets ethical standards for child participants; procurement and deployment questions do not arise.

Public procurement. For school and district procurement, E-NALD compliance should be integrated into tendering and contracting, leveraging market incentives to encourage vendor compliance (Institute of Electrical and Electronics Engineers, 2021). Intensive capacity building is needed to address weak procurement processes and information asymmetries in decentralized education systems, and procurement templates based on the IEEE P7000 series and the UNESCO 2025 Recommendation can lower adoption barriers (United Nations Educational, Scientific and Cultural Organization, 2025; Institute of Electrical and Electronics Engineers, 2021). Regulatory responses to school facial recognition in the United Kingdom (Andrejevic and Selwyn, 2020) and Finland’s national AI strategy (Finnish Ministry of Economic Affairs and Employment, 2017) offer transferable structures combining mandatory impact assessments with independent oversight (Andrejevic and Selwyn, 2020; Finnish Ministry of Economic Affairs and Employment, 2017). Regulatory sandboxes have been proposed as a complementary mechanism for testing neurotechnologies under supervisory conditions before wider deployment (Johnson, 2024).

Commercial deployment. For vendors bringing BCI products to market, technical standards development organizations (ISO, IEC, IEEE) should incorporate E-NALD requirements as normative elements (Institute of Electrical and Electronics Engineers, 2021), and national regulators should issue BCI-specific guidance based on E-NALD. Under the EU AI Act, whether a BCI attracts high-risk obligations depends on whether it constitutes an “automated assessment” under Annex III (European Parliament and Council of the European Union, 2024); the boundary between “limited-risk” (transparency-only) and “high-risk” (consequential pedagogical decisions, such as special education placement) is a function of system use. Rule A1 accuracy benchmarks, Safeguard A2 bias audits, and Rule E1 outcome validation provide pragmatic empirical indicators for this risk classification. A governance sandbox offers a transitional mechanism in which selected vendors operate with relaxed compliance for 12–18 months under enhanced monitoring and required accuracy and bias reporting (United Nations Children’s Fund, 2021), extending the PDPC Singapore AI sandbox (Personal Data Protection Act, 2012) and drawing on UK ICO regulatory guidance (Information Commissioner’s Office, 2021); success metrics include improved classifier accuracy for underrepresented demographic groups, reduced misclassification leading to inappropriate interventions, and positive feedback from teacher-student trials.

Routine school use. For systems already deployed in classrooms, the critical safeguards are human-facing rather than technical: training in BCI technical fundamentals, pedagogical interpretation of cognitive-state outputs, and override authority (Rule D2), which is essential both for effective deployment and for preventing teacher disengagement or technology rejection (Khulbe and Tammets, 2023). In this context the oversight actors are schools and education authorities, and the governing question is whether teachers retain meaningful pedagogical judgment and students retain meaningful agency over their learning.

### 5.2 Neurodiversity and equitable access

A dimension largely absent from neurotechnology governance discussions is the interaction between BCI systems and neurodivergent learners. In most developed countries, 15–20% of the school-age population has ADHD, autism spectrum disorder (ASD), dyslexia, or other neurodevelopmental conditions (American Psychiatric Association, 2022). Well-designed adaptive learning technologies can provide personalized accommodations difficult to offer in standard classroom settings; poorly designed systems can harm both neurotypical and neurodivergent learners by misinterpreting neural differences as evidence of disinterest or inability (Mecacci and Haselager, 2019; Sullivan et al., 2018). E-NALD addresses neurodiversity through four mechanisms. Under the Bias Audit Rule (Section 4.3), accuracy metrics must be disaggregated by neurodevelopmental status, with systems showing significant accuracy disparities subject to remediation or deployment restrictions. This requirement applies not only to officially diagnosed individuals—the 15–20% figure (American Psychiatric Association, 2022) counts only diagnosed learners, while many undiagnosed differences or subclinical variations may similarly impact BCI performance. The Bias Audit Rule should therefore extend to a “suspected underperformance” category triggering assessment when accuracy gaps are observed regardless of formal diagnosis. Second, vendors should be required to disclose training data demographics including neurodevelopmental representation. Third, educational BCIs should incorporate individual calibration procedures, as some learners’ neural signals may differ from population norms and group classifiers may systematically misclassify such signals (Mecacci and Haselager, 2019). Fourth, schools should conduct “neurodiversity impact assessments” before BCI procurement to evaluate whether system training data, validation processes, and calibration options are adequate for their student population’s neurodevelopmental composition.

### 5.3 Global south considerations

E-NALD’s legal and regulatory references primarily derive from Western jurisdictions (European Union, United States) and international organizations headquartered in the global North. We explicitly acknowledge these limitations. Educational technology markets are expanding rapidly in Asia, Africa, and Latin America UNESCO (2023), where governance capacity may lag behind public enthusiasm for neurotechnology, and where E-NALD’s complex compliance demands may be achievable only in well-resourced educational systems. Transferability is complicated by varying cultural attitudes toward technology and childhood, diverse institutional structures for data protection and procurement, and substantial compliance costs. E-NALD compliance is not costless: hardware alone for a 50-student deployment—50 consumer EEG headsets retailing at roughly US$500–600 each the BrainCo Focus headband was priced at 3,499–4,299 yuan, approximately US$500–625, on major Chinese retail platforms in 2019 (People’s Daily Online, 2019)—already amounts to approximately US$25,000–30,000 before software licensing, teacher training, or audit costs. Cultural contexts legitimately shape how safeguards are implemented: while Western frameworks emphasize individual consent and privacy, other cultures may weigh community benefit or collective outcomes more heavily. Participatory processes are needed to enable local stakeholders to shape implementation within the universal floor. We propose three strategies for adapting E-NALD to diverse global contexts. First, we have restructured the tiered approach so that resource constraints affect the pace of full implementation, never the level of protection: all 15 safeguards constitute a universal ethical floor for any deployment involving children, and what varies by context is the sequence in which full compliance is achieved. Jurisdictions with limited governance capacity should begin with Phase 1 safeguards—S1 (Child-Specific Consent), S2 (Data Minimization), and S3 (Purpose Limitation)—because these are prerequisites for any ethical deployment and are also the least resource-intensive to satisfy; Phase 2 (A1-A3, D1-D4, I1-I2) and Phase 3 (E1-E3) safeguards should follow as institutional capacity develops, with external support where needed. Phased implementation is a scheduling commitment, not a derogation: no safeguard is optional, and no resource context justifies relaxing fundamental protections for children. Second, international bodies such as UNESCO and UNICEF should provide capacity-building support for developing countries adopting neurotechnology (United Nations Children’s Fund, 2024; United Nations Educational, Scientific and Cultural Organization, 2025). Third, regional adaptation should be encouraged—for example, an Asia-Pacific framework reflecting local cultural and institutional contexts. Singapore’s Personal Data Protection Act 2012 (2020 Revised Edition) (Personal Data Protection Act, 2012) provides data protection obligations generally aligned with E-NALD’s Data Minimization and Purpose Limitation principles, and Singapore’s regulatory sandbox mechanism for emerging technologies provides a foundation for incremental E-NALD application.

### 5.4 Comparative positioning of the E-NALD framework

Table 2 compares E-NALD against two existing governance instruments-the UNESCO 2025 Recommendation and the EU AI Act-with UNICEF policy guidance used as a supplementary soft-law reference point.

## 6 Discussion

### 6.1 Neurorights: engagement with critical perspectives

The E-NALD framework can be understood within the context of the “neurorights” debate. Proponents including Yuste et al. (2017), Ienca and Andorno (2017), and Ienca (2021) have called for novel fundamental rights of the brain and mind: cognitive liberty (the right to make decisions regarding one’s own brain), mental privacy (the right against unauthorized neural data access), mental integrity (the right to protection against harmful brain and mind manipulation), and psychological continuity (the right to protection against changes in personal identity). These proposed rights have been adopted at the national level in Chile and Colorado and have informed the UNESCO 2025 Recommendation and Council of Europe deliberations (Yuste et al., 2017; Ienca and Andorno, 2017). Chile’s 2021 constitutional reform enacted the right to “neuronal integrity,” broadly prohibiting neural data use and neural activity manipulation without informed consent. Colorado Senate Bill 24–205 mandates neurodata collection transparency and grants individuals access, correction, and deletion rights for neural information. Significant criticisms have been raised. Blitz and Bublitz (2021) question whether neural data warrants special legal treatment, suggesting existing data protection frameworks may suffice and that “neuro-exceptionalism” risks inappropriately elevating brain-based information. Gilbert and Russo (2023) warn against “speculative ethics”—anticipatory measures taken ahead of actual capabilities. Hertz (2023) questions whether new neurorights are needed at all, arguing that the established right to freedom of thought may already protect the interests at stake. The E-NALD framework occupies a position between these perspectives (Jobin et al., 2019). Its focus on currently deployed, commercially available technologies rather than speculative future scenarios avoids “speculative ethics” concerns. Its ethical precepts are grounded in established concepts of child protection, data protection, and consumer safety rather than novel rights claims, making the framework compatible with either neurorights-supportive or neurorights-skeptical legal foundations (United Nations Educational, Scientific and Cultural Organization, 2025; Institute of Electrical and Electronics Engineers, 2021). Simultaneously, the framework recognizes that neural data exhibits distinctive features—pre-cognitive sensitivity, capacity to reveal extensive information without the data subject’s awareness, and stability as a biometric identifier (Ienca et al., 2018)—that may warrant protections extending beyond standard information protection. Risks addressed by the Bias Audit Rule and Manipulation Prohibition Rule are specific to neural monitoring and have been documented in clinical BCI scenarios (Yuste et al., 2017) that standard data protection does not adequately capture.

### 6.2 From educational BCIs to neurorobotic governance: broader implications

While this paper focuses on educational BCIs as a specific application domain, the E-NALD framework’s contributions extend to neurorobotic governance more broadly. Educational BCIs represent a paradigmatic case for neurorobotic governance for several reasons: they involve sustained, long-term deployment of embodied autonomous neural systems in captive user populations; they process particularly sensitive biological data (neural signals) from vulnerable child populations; they operate in institutional contexts (schools) with inherent power asymmetries; and they make consequential decisions affecting fundamental rights (education, cognitive liberty, mental integrity). These characteristics make educational BCIs a stress test for neurorobotic governance principles. Whether safeguards that prove necessary and feasible in this demanding context transfer effectively to less constrained neurorobotic applications is an open empirical question that we cannot settle here; we advance the transferability claim as a plausible, testable conjecture rather than a demonstrated result. The framework’s stage-based architecture (Sense-Analyze-Decide-Intervene-Evaluate) maps naturally onto the control pipelines of diverse neurorobotic systems, from prosthetic devices to wearable cognitive enhancement systems to autonomous robots driven by brain-inspired algorithms. Each stage presents characteristic governance challenges that transcend specific application domains: signal acquisition raises privacy and consent issues; algorithmic analysis raises accuracy, bias, and explainability concerns; decision-making raises autonomy and human oversight questions; intervention raises manipulation and harm prevention challenges; and evaluation raises data retention and outcome validation requirements. By developing granular governance mechanisms for each stage, the E-NALD framework offers a candidate template—hypothesis-generating rather than validated—for sector-specific governance adaptation across the neurorobotics landscape; any such adaptation would require domain-specific validation of both the stages and the safeguards. Furthermore, the Governance by Design approach—embedding ethical requirements into technical architecture and institutional processes—offers a generalizable strategy for addressing the “implementation gap” that characterizes much current AI and robotics ethics discourse. The integration of enforceable standards, lifecycle coverage, and sector-specific operationalization that distinguishes GbD from predecessor frameworks responds to governance challenges common across embodied autonomous systems. As neurorobotic technologies transition from research prototypes to commercial products deployed in everyday environments, governance frameworks must evolve correspondingly. E-NALD illustrates one candidate pathway for this evolution, whose generalizability remains to be established through cross-domain application.

### 6.3 The ethics-regulation relationship: beyond the implementation gap

Changing technologies and evolving social understandings require an iterative relationship between ethical principles and research ethics governance safeguards, with continuous translation and adaptation. United Nations Educational, Scientific and Cultural Organization (2025) and Organisation for Economic Co-operation and Development (2019) represent pioneering initiatives consolidating neuroethics principles into widely accepted soft law instruments. However, the specifics of creating operational safeguards from principles require ongoing collaboration among affected parties, ethicists, technologists, and regulators, as Black (2002), Yuste et al. (2017), and Ienca and Andorno (2017) have observed, Stilgoe et al. (2013), and Schermer (2011) have noted more broadly in technology governance. The E-NALD framework contributes to this iterative process in three ways: by providing a sector-specific translation mechanism from abstract principles to concrete safeguards; by offering a structured template for similar translations in other neurotechnology domains; and by grounding its safeguards in existing governance frameworks, thereby reducing institutional barriers to implementation.

### 6.4 Children’s rights in the Neurotechnology age

The E-NALD systematic approach corresponds to the three core principles of the CRC (Lansdown, 2005; United Nations Children’s Fund, 2024). Article 3’s “best interests of the child” principle underlies the Non-Coercion, Manipulation Prohibition, and Outcome Validation Rules. The Child-Specific Consent Rule (Lansdown, 2005) reflects the evolving capacities principle (Article 5). Operationalization of the right to be heard (Article 12) is achieved through the Consent Rule and Right to Disconnect. There is growing international consensus that children deserve enhanced safeguards regarding neurotechnology, as reflected in the United Nations Human Rights Council (2024), Naufel and Klein (2020), Council of Europe (2024), and United Nations Educational, Scientific and Cultural Organization (2025). The E-NALD framework contributes to this emerging normative space by adding the operational specificity these instruments currently lack, transforming protection intentions into concrete technical and institutional requirements for neurorobotic systems deployed in educational environments.

## 7 Conclusion

This paper has presented Governance by Design (GbD) as a research ethics governance philosophy and the Ethical NALD (E-NALD) framework as a sector-specific implementation for educational brain-computer interfaces—a rapidly evolving class of neurorobotic systems with significant implications for children’s rights and societal values. Three key contributions are offered. First, recognizing that the Ethics by Design paradigm has been applied to technologies for some time, we have extended it with enforceable ethical requirements essential when these technologies are deployed in compulsory settings with vulnerable populations. We have demonstrated that Governance by Design should not be considered an alternative to Ethics by Design, but rather as a synthesizing extension incorporating enforcement mechanisms, lifecycle coverage, and sector-specific operationalization. Second, we have shown that the NALD model can identify intervention points in the neuroadaptive learning pipeline that give rise to unique ethical governance concerns addressable through targeted safeguards at each stage: Sense, Analyze, Decide, Intervene, and Evaluate. Third, we have operationalized internationally established neuroethics principles into concrete, law-based safeguards grounded in the UNESCO 2025 Recommendation on the Ethics of Neurotechnology, the EU AI Act, the UN Convention on the Rights of the Child, and other foundational instruments. By situating educational BCIs within the broader context of neurorobotics and embodied autonomous neural systems, this study illustrates how governance frameworks for such systems must account for their unique technical characteristics—closed-loop signal processing, real-time adaptive intervention, and sustained intimate data collection—while remaining grounded in established principles of human rights and child protection. The E-NALD framework offers a candidate template for this integration; its adaptation to other neurorobotic application domains, which we encourage, requires domain-specific validation of the stages, safeguards, and thresholds. Several limitations are acknowledged. First, the framework is conceptual and its safeguards and thresholds require validation through pilot studies. Second, the legal references emphasize Western jurisdictions and require adaptation to global contexts. Third, advancing BCI technology may necessitate periodic revision of accuracy thresholds and other parameters. Three directions for future research are proposed. First, longitudinal analysis of E-NALD implementation at the school district level would yield valuable data on safeguard effectiveness, compliance burdens, and framework operation across diverse educational settings. Second, comparative studies of framework application across jurisdictions—particularly between Europe, North America, and Asia-Pacific—would illuminate transferability across legal and cultural boundaries. Third, as neuroadaptive technology evolves, regular reevaluation of system accuracy will be necessary to ensure E-NALD thresholds remain appropriate. As educational BCIs transition from pilot projects to broader implementation, governance frameworks must evolve correspondingly. Governance by Design provides a principled approach to responsible innovation that safeguards children’s rights while preserving the potential of neuroadaptive technologies as pedagogical tools.

## Data Availability

The original contributions presented in the study are included in the article/supplementary material, further inquiries can be directed to the corresponding author/s.
